# Outcomes from a three-arm randomized controlled trial of frequent immersion in thermoneutral water on cardiovascular risk factors

**DOI:** 10.1186/s12906-016-1241-7

**Published:** 2016-07-27

**Authors:** Johannes Naumann, Catharina Sadaghiani, Nina Bureau, Stefan Schmidt, Roman Huber

**Affiliations:** 1 Interdisciplinary Center for Treatment and Research in Balneology, Institute for Environmental Health Sciences and Hospital Infection Control, Medical Faculty, Medical Center University of Freiburg, Breisacher Straße 115b, Freiburg im Breisgau, 79106 Germany; 2 Department of Psychosomatic Medicine, Medical Faculty, Medical Center University of Freiburg, Hauptstraße 8, Freiburg im Breisgau, 79104 Germany; 3 Center for Complementary Medicine, Institute for Environmental Health Sciences and Hospital Infection Control, Medical Faculty, Medical Center University of Freiburg, Breisacher Straße 115b, Freiburg im Breisgau, 79106 Germany; 4 Institute of Transcultural Health Studies, European University Viadrina Frankfurt (Oder), Grosse Scharrnstr. 59, 15230 Frankfurt (Oder), Germany

**Keywords:** Cardiovascular, Hypertension, Balneotherapy, Hydrotherapy, Immersion

## Abstract

**Background:**

Cardiovascular diseases are a main cause of mortality worldwide. Spa bathing and immersion in thermoneutral water (ITW) have a long history in the treatment of cardiovascular risk factors.

**Methods:**

We conducted a three-arm parallel-group, randomized controlled study to investigate the effects of frequent ITW on moderately elevated blood pressure (BP). Here, we report on the secondary outcomes, i.e. the influence of immersion in thermoneutral water on further cardiovascular risk factors: body mass index (BMI), waist circumference, blood lipids, fasting blood glucose and C-reactive protein. Patients (age 57.6 ± 9.6 years, BMI 29.5 ± 5.7 kg/m^2^) with mild to moderately elevated BP received ITW for 45–60 min in pools of thermal-mineral water at 34.0–36.0 °C four times a week for 4 weeks. One group (Bath1) reduced the intensity to one bath a week for a further 20 weeks, while the other group (Bath2) continued bathing four times a week. The control group was instructed to relax for 45–60 min four times a week for 24 weeks using a relaxation CD.

**Results:**

The secondary analysis of the intention-to-treat population (*N* = 59) did not show a significant reduction of BMI, waist circumference, blood lipids, fasting blood glucose or C-reactive protein in patients with a mild to moderately elevated BP after 4 and 24 weeks ITW, respectively. There were no significant differences between the groups.

**Conclusion:**

Thus, we did not find evidence to support our ITW program being an efficacious intervention to induce cardiovascular alterations in this population of hypertensive patients.

**Trial registration:**

DRKS00003980 at drks-neu.uniklinik-freiburg.de, German Clinical Trials Register (registration date 2012-07-10).

**Electronic supplementary material:**

The online version of this article (doi:10.1186/s12906-016-1241-7) contains supplementary material, which is available to authorized users.

## Background

Atherosclerotic cardiovascular disease (CVD) remains the major cause of premature death in Europe, even though CVD mortality has fallen considerably over recent decades in many European countries. CVD is strongly associated with lifestyle, especially the use of tobacco, unhealthy diet habits, physical inactivity and psychosocial stress. The World Health Organization (WHO) has stated that over three-quarters of the deaths from CVD could be prevented with adequate changes in lifestyle [1]. However, adherence in real life to lifestyle modifications is poor, with fewer than 50 % of patients practising any form of modification at all [2, 3]. Thus, there is a strong need to find treatment options that lead to better adherence to lifestyle changes. With its long lasting history, both in the Eastern and Western hemispheres, immersion in thermoneutral water (ITW) or spa bathing may provide a treatment opportunity that meets wide acceptance and availability [4–6].

During ITW, water pressure causes short-term cardiovascular responses as blood shifts from the legs and abdomen to the right atrium of the heart. ITW induces an increase in stroke volume, a reduction of heart rate, an increase of cardiac output, and a reduction of total peripheral vascular resistance [7–9]. Furthermore, head-out ITW has several advantages: Water reduces weight bearing because of buoyancy, and water resistance strengthens the muscles. In combination with aquatic exercises, it provides obese and overweight people with a noninjurious and easy to learn opportunity to lose weight [10]. There is clinical evidence for a large antihypertensive effect from exercise in thermoneutral water [11, 12], and it is particularly important for lifestyle disease prevention as well as long-term care prevention, and is prevalent among the middle-aged and elderly population [13]. Balneotherapy and especially spa therapy have also been studied for their possible metabolic and anti-inflammatory effects, however the results are inconsistent and scientific evidence is limited [14–18].

We therefore wished to address the question of whether the positive effects on cardiovascular risk factors (CVRF) are mainly due to ITW or to the exercise and other components of spa therapy. We have previously reported on the primary outcome of this study, which did not show a significant reduction of blood pressure (BP) in patients with mild to moderately elevated BP after 4 and 24 weeks ITW [19]. Here, we report on the secondary outcomes. We assumed that frequent and regular use of ITW reduces CVRF in patients with hypertension grade 1 and 2.

## Methods

### Study design and participants

This study is reported according to the CONSORT guidelines for the presentation of clinical trials [20]. This randomized, controlled, three-arm parallel-group study was performed at the Interdisciplinary Center for Treatment and Research in Balneology in Southern Germany. Participants were recruited via announcement in local newspapers and by sending written information about the study to local physicians, hospitals and pharmacies. Participants were eligible for inclusion if aged ≥ 18 years, had a diagnosis of arterial hypertension with an office systolic BP (SBP) of 140–180 mm Hg, and diastolic BP (DBP) of 90–110 mm Hg (grade 1 and 2 hypertension according to ESH/ESC guidelines 2013 [21]), had no change of antihypertensive medication in the last four weeks and no indication to change medication within the next four weeks. Exclusion criteria were severe disease - heart failure grade 3 or 4, peripheral arterial disease grade 3 or 4, unstable angina, renal failure, creatinine > 1.5 mg/dl - that might limit the patient’s capacity to participate in the study or make other treatments necessary, secondary hypertension, acute infection, fever, reasons against use of public baths such as open wounds, ITW (>1 per week) in the past two months, pregnancy and participation in other interventional studies. Written informed consent was obtained from all the participants before inclusion; ethical approval was given by local Ethics Committee (Ethics-Commission Medical Center University of Freiburg; No. 111/12). Clinical trial authorization was obtained from the Federal Institute for Drugs and Medical Devices (BfArM, Germany) and the Regional Council Freiburg. The study was prospectively registered in the German Clinical Trials Register (DRKS00003980) and conformed to the principles of the Declaration of Helsinki and to the GCP guidelines of the European Community.

### Interventions

After completion of all baseline measurements participants were randomly allocated to one of two intervention groups (Bath1, resp. Bath2) or the control group (relaxation). Both bathing groups started ITW (34.0–36.0 °C) for 45–60 min four times a week for four weeks. Treatment locations were three spa centers in South-West Germany (Bad Krozingen, Freiburg and Bad Bellingen) with a comparable water mineral content (see Additional file 1: Table S1) and temperature. All participants were instructed to have head-out ITW (minimum deeper than axilla). After 4 weeks, one bathing group (Bath2) continued with four baths a week for another 20 weeks, while the other bathing group reduced to one bath a week (Bath1). The control group was advised to practice relaxation at home four times a week for 45–60 min for 24 weeks. Patients were provided with a relaxation CD, teaching them progressive muscle relaxation according to Jacobson, with some elements of awareness produced by RH to treat his patients. All groups were given written information on non-pharmacological methods to reduce BP edited by [22] and were advised to follow the instructions.

### Outcome measures: CVD parameters and blood biochemistry

The primary outcome was BP measured after 4 and 24 weeks ITW, respectively, and has been reported previously [19]. Here, we report on the secondary clinical outcomes of CVRF: BMI, waist circumference, blood lipids, fasting blood glucose and C-reactive protein (CRP). Body weight was measured by using a digital scale (Soehnle professional, no. 7701; self-calibrating), height was measured by using a telescopic mechanical height rod (Seca, no. 2251721009), waist circumference was measured at the umbilical level using an inelastic measuring tape on the bare skin and recorded to the nearest 0.1 cm. The tape was snugged horizontally around the abdomen passing across the umbilicus without causing compression on the skin. Measurement was performed at the end of normal expiration. Blood samples were collected for total cholesterol, high-density lipoprotein (HDL) cholesterol, low-density lipoprotein (LDL) cholesterol, triglyceride and CRP (all measured in serum, centrifuged 30 min after taking blood) and fasting blood glucose (NaF-Plasma). Analyses were done at the Institut für Klinische Chemie und Laboratoriumsmedizin (IKCL), Medical Center University of Freiburg (certified by Deutsche Akkreditierungsstelle DAkkS according to ISO 9001:2008).

### Randomization and blinding

Randomization codes (single block of 60 patients) were generated by an independent statistician (SS). Because several participants withdrew consent after randomization but before being informed about their allocation, a further six participants were randomized in a single block. Enrolment (JN) was done directly after informed consent was obtained from the patients. Study IDs were assigned chronologically. In order to conceal allocation, SS was informed and added the study ID in chronological order in the randomization list. SS had no interaction at all with the patients. On their next visit, patients received a sealed, opaque envelope informing them of their allocated treatment.

### Statistical analyses and sample size

Efficacy parameters were analyzed based on the intention-to-treat (ITT) population, defined as all allocated participants, applying the last-observation-carried-forward (LOCF) approach to impute missing data. Baseline differences between the three groups were assessed by either univariate analyses of variance (ANOVA) or by Chi^2^ tests. To compare the effectiveness of ITW, a one-way between-groups analysis of covariance (ANCOVA) was conducted. The analysis model was built with group allocation as the independent variable, BMI, waist circumference, blood lipids, fasting blood glucose and CRP as the dependent variables, and the corresponding baseline scores as covariates. Prior to the analysis, we made sure that the specific assumptions for normality and homogeneity of variance for the one-way ANCOVA were met.

We report *P*-values and the respective partial eta^2^ as effect size. A sample size calculation was performed for the primary outcome BP and resulted in 20 patients per group. Analyses were performed using SPSS version 20 (SPSS, Chicago IL). Data management and analyses were performed blinded to treatment allocation (SS, TW).

## Results

### Study population

**Fig. 1:**
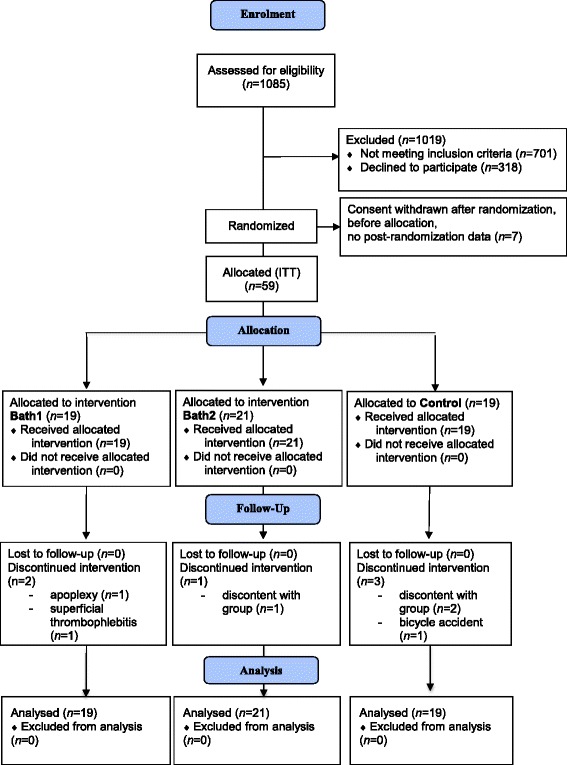
CONSORT 2010 Flow Diagram

Details of participant flow are given in the figure (Fig. 1). During enrolment between July 2012 and April 2013, we screened 1085 patients. Of this number, 1019 participants were excluded - 701 for not fulfilling the inclusion criteria (mainly not having elevated BP values) and 318 for various other reasons such as not accepting randomization to regular baths or no baths for 6 months. In the end, a total of 66 participants met the inclusion criteria and were randomized into the study. Of these, seven withdrew their consent before receiving their allocation and were excluded from ITT-analysis, resulting in a final sample of 59 participants. According to Fergusson et al. [23], this so-called post-randomization exclusion from the primary analysis may be legitimate if patients have not received the intervention. In this case, excluding patients does not introduce bias and may lead to a more informative analysis.

Baseline details for the 59 participants allocated into the study are shown in Table 1. All participants were of Caucasian background, mostly overweight (BMI 29.5 ± 5.7 kg/m^2^) and medium age was 57.6 ± 9.6 years. Thirty-three patients (55.9 %) were taking antihypertensive medication (i.e. ACE inhibitors, AT_1_-receptor antagonists, diuretics, Calcium-antagonists) see Additional file 2: Table S2. There were no statistically significant differences at baseline between the study groups in age, BMI, BP or CVRF. There were statistically significant differences at baseline between the study groups in fasting blood glucose (Bath1: 110, Bath2: 90, Control: 95 mg/dl; *P*_*Bath1 x Bath2*_ = 0.02), gender (*P*_*Bath1 x Bath2*_ = 0.04), and the use of antihypertensive medication (Bath1: 57.9 %, Bath2: 33.3 %, Control: 78.9 %; *P*_*Bath2 x Control*_ = 0.02) (for details see Additional file 2: Table S2).

**Table 1:** Baseline demographic and clinical characteristics of the intention-to-treat population (*N* = 59)

	Overall *N* = 59	Bath1 *N* = 19	Bath2 *N* = 21	Control *N* = 19	*P*
Gender (female/male)	29/30	5/14	14/7	10/9	0.04*^a^
Age (years)	57.6 (9.6)	60.2 (6.7)	57.1 (10.0)	55.5 (11.4)	0.32
Patients taking antihypertensive medication (n)	33 (55.9 %)	11 (57.9 %)	7 (33.3 %)	15 (78.9 %)	0.02*^a^
No CVRF (*n*)	2	0	2	0	0.46^a^
1–2 CVRF (*n*)	32	9	13	10	
≥3 CVRF (*n*)	22	9	5	8	
CVD (*n*)	3	1	1	1	
Smoking status (smoker/non-smoker)	9/50 (15 %)	1/18 (5 %)	4/17 (19 %)	4/15 (21 %)	0.33^a^
SBP (mm Hg)	145.2 (10.2)	147.2 (7.8)	143.8 (12.3)	144.8 (9.9)	0.55
DBP (mm Hg)	90.1 (8.5)	91.8 (8.4)	90.5 (9.9)	88.1 (7.0)	0.42
BMI (kg/m^2^)	29.5 (5.7)	29.9 (5.3)	27.4 (5.3)	31.3 (5.9)	0.08
Waist circumference (cm)	102.0 (13.0)	106.0 (14.0)	98.0 (13.0)	103.0 (11.0)	0.20
Cholesterol total (mg/dl) (50–200)	226.0 (36.0)	219.0 (38.0)	230.0 (35.0)	227.0 (36.0)	0.63
LDL (mg/dl) (<160)	138.0 (33.0)	135.0 (33.0)	143.0 (31.0)	137.0 (35.0)	0.72
HDL (mg/dl) (>40)	48.0 (15.0)	47.0 (13.0)	51.0 (15.0)	44.0 (15.0)	0.28
LDL/HDL Quotient (<3)	3.2 (1.3)	2.9 (0.9)	3.1 (1.5)	3.5 (1.5)	0.38
Triglyceride (mg/dl) (<150)	153.0 (88.0)	168.0 (107.0)	126.0 (56.0)	168.0 (95.0)	0.22
CRP (mg/l) (0–5)	2.7 (2.3)	2.5 (2.3)	2.3 (2.3)	3.3 (2.3)	0.36
Fasting blood glucose (mg/dl) (70–110)	99.0 (23.0)	110.0 (30.0)	90.0 (18.0)	95.0 (15.0)	0.02*

Data are shown as mean and standard deviation (SD)

*P < 0.05

*BMI* body mass index, *CVRF* cardiovascular risk factors, *CVD* cardiovascular disease, *SBP* systolic blood pressure, *DBP* diastolic blood pressure, *CRP* C-reactive protein

^a^Chi^2^-test

### Safety, tolerability and compliance

Six (10.2 %) participants dropped out during the study, two in Bath1, one in Bath2 and three in the control group. The alleged reasons for leaving the study were discontent with the assigned group (*n* = 3) and adverse events. In group Bath1 there was one serious adverse event (apoplexy that occurred in a patient with preexisting cerebral apoplexy who had reduced the frequency of baths) and one adverse event (superficial thrombophlebitis). After a bicycle accident one participant from the control group discontinued the study. In any case there was no association between drop-out and treatment (ITW/relaxation).

A further 34 non-treatment-related adverse events were reported by 25 patients - 20 common colds, one nasal bleeding, two headaches, one lumbago, three gastrointestinal disturbances, one cystitis, one sore throat, one gastric ulcer, one temporary itching of the legs, two cases of feeling unwell without specific complaints and one atrial fibrillation that was converted into sinus rhythm with bisoprolol. Two changes in antihypertensive treatment were recorded. One participant stopped medication (ramipril), and one (with new diagnosis of atrial fibrillation) started medication (bisoprolol). Adherence to therapy (Table 2) was good, with 77 % of the participants taking more than 75 % of the recommended number of baths in the intense bathing group (Bath2) and 89 % in the less intense bathing group (Bath1). None of the patients were lost to follow-up.

**Table 2:** Adherence to therapy and bath frequency

		*n*	Frequency Target	Frequency Actual	Adherence (%)	>75 % Adherence (*n*)
Week 1–4	Bath 1	19	16	15	94	
	Bath 2	21	16	14	88	
Week 5–24	Bath 1	19	20	18	90	
	Bath 2	21	80	57	71	
Total	Bath 1	19	36	32	89	16
	Bath 2	21	96	74	77	9

Week 1–4: bathing four times a week (Bath 1, Bath 2)

Week 5–24: bathing once a week (Bath 1); bathing four times a week (Bath 2)

### Data monitoring

Data monitoring was done blind to treatment allocation, with double data entry by two independent persons for 20 % of the values (revealing one error in 1776 data cases).

### Effects of treatment on CVRF

Table 3 shows the effects of head-out ITW (Bath1, Bath2) or relaxation (Control) on BMI, waist circumference, blood lipids, fasting blood glucose and CRP. The mean (SD) BMI at baseline was 30 (5) (Bath1), 27 (5) (Bath2) and 31 (6) (Control). After 24 weeks, corresponding values were 29 (5) (Bath1), 27 (6) (Bath2) and 31 (6) (Control) (*P* = 0.30, partial eta^2^ 0.04). No significant differences were observed between the groups in waist circumference, blood lipids, fasting blood glucose and CRP. At the end of treatment one out of nine smokers (11 %) had stopped smoking (Bath1), two had reduced (Bath2 *n* = 1; Control *n* = 1).

**Table 3:** Effect of ITW on cardiovascular risk factors (CVRF) after 24 weeks; mean (SD), intention-to-treat population (*N* = 59)

CVRF	Group	Baseline	Week 4	Week 24	Change Baseline/Week 24	*P* Value	Partial eta^2^
BMI (kg/m^2^)	Bath 1	29.9 (5.3)	29.5 (5.0)	29.3 (4.9)	−0.6 (3.2)		
	Bath 2	27.4 (5.3)	27.5 (5.5)	27.2 (5.8)	−0.2 (3.5)	0.30	0.04
	Control	31.3 (5.9)	31.5 (5.9)	31.1 (5.7)	−0.2 (3.7)		
Waist circumference (cm)	Bath 1	105.5 (13.6)	103.7 (13.8)	104.1 (12.8)	−1.4 (8.4)		
	Bath 2	98.4 (12.7)	98.9 (14.6)	98.2 (15.6)	−0.2 (9.4)	0.69	0.01
	Control	102.5 (10.7)	102.3 (9.9)	101.8 (10.7)	−0.7 (6.8)		
Cholesterol total (mg/dl) (50–200)	Bath 1	219.4 (38.2)	215.2 (32.3)	211.1 (34.6)	−8.3 (23.3)		
	Bath 2	230.2 (35.0)	233.1 (32.4)	236.6 (47.3)	6.4 (28.5)	0.09	0.09
	Control	226.9 (35.6)	212.9 (33.3)	222.2 (34.4)	−4.7 (22.2)		
LDL (mg/dl) (<160)	Bath 1	134.5 (33.3)	135.7 (29.1)	129.9 (27.0)	−4.6 (20.0)		
	Bath 2	143.0 (30.6)	144.9 (25.7)	147.2 (32.7)	4.2 (20.1)	0.13	0.09
	Control	136.8 (35.1)	128.9 (34.2)	141.7 (34.3)	4.9 (22.0)		
HDL (mg/dl) (>40)	Bath 1	47.3 (13.1)	47.0 (15.3)	45.8 (11.0)	−1.5 (7.9)		
	Bath 2	51.4 (14.7)	52.8 (14.2)	51.1 (15.1)	−0.3 (9.4)	0.84	0.01
	Control	44.0 (15.2)	45.8 (13.6)	45.9 (15.5)	1.9 (9.7)		
LDL/HDL Quotient (<3)	Bath 1	2.9 (0.9)	3.1 (1.0)	3.0 (0.9)	0.1 (0.6)		
	Bath 2	3.1 (1.5)	2.9 (0.9)	3.0 (1.0)	−0.1 (0.9)	0.39	0.04
	Control	3.5 (1.5)	3.1 (1.4)	3.6 (1.9)	0.1 (1.1)		
Triglyceride (mg/dl) (<150)	Bath 1	168.0 (107.0)	146.0 (77.0)	126.0 (42.0)	−42.0 (77.6)		
	Bath 2	126.0 (56.0)	134.0 (66.0)	121.0 (56.0)	−5.0 (35.4)	0.77	0.01
	Control	168.0 (95.0)	149.0 (68.0)	144.0 (57.0)	−24.0 (60.1)		
CRP (mg/l) (0–5)	Bath 1	2.5 (2.3)	1.6 (1.3)	1.6 (1.3)	−0.9 (1.5)		
	Bath 2	2.3 (2.3)	1.8 (1.9)	2.3 (2.7)	0.0 (1.6)	0.64	0.02
	Control	3.3 (2.3)	2.5 (2.3)	2.4 (2.5)	−0.9 (1.5)		
Fasting blood glucose (mg/dl) (70–110)	Bath 1	110.0 (30.0)	101.0 (24.0)	104.0 (26.0)	−6.0 (18.1)		
	Bath 2	90.0 (18.0)	91.0 (12.0)	88.0 (8.0)	−2.0 (12.6)	0.55	0.03
	Control	95.0 (15.0)	90.0 (15.0)	93.0 (15.0)	−2.0 (9.5)		

Analysis of covariance (ANCOVA) with baseline values as covariates. *P*-value and partial eta^2^ refer to time × group interaction

*BMI* body mass index, *CRP* C-reactive protein, *HDL* high-density lipoprotein, *ITW* immersion in thermoneutral water, *LDL* low-density lipoprotein

## Discussion

Compared to a control group using regular relaxation techniques, intense ITW (one or four times a week) over a 24-week period did not reduce CVRF after either 4 or 24 weeks, respectively. This result was unexpected and several possibilities merit discussion to explain our findings.

### Intensity

The intensity of ITW used does not explain our negative results, because we investigated high and low intensity ITW.

### Temperature

High temperature (≥37.0 °C) causes more vasodilation and enhances metabolism, the temperature of the water (34.0–36.0 °C) may therefore have been too low. However, there is only little evidence for a cardiovascular influence and the results are inconsistent. In a small trial with eight patients with diabetes mellitus type 2 [24] bathing in hot tubs at 38.0–40.0 °C for 30 min per day, six times per week for three weeks resulted in a mean weight loss of 1.7 kg, and a decrease of fasting blood glucose from 182 mg/dl to 159 mg/dl. A Japanese RCT using hot baths (40.0 °C; 20 min once a fortnight) found no significant effect on BMI, waist circumference, serum lipids or blood glucose in healthy subjects [25]. A Hungarian study found no significant reduction in plasma lipids or CRP in 42 ambulatory patients with degenerative musculoskeletal disease after 15 baths at 38.0 °C over 3 weeks compared to baseline [16].

### Exercise

It may also be necessary to combine ITW (32.0 °C) with exercise. This was shown in a 12-week randomized controlled trial in which a large significant reduction of BP (office SBP −36 mm Hg, DPB −12 mm Hg; 24-h ambulatory SBP −17 mm Hg, DBP −9 mm Hg) was observed in patients with resistant hypertension [12]. In a 10-week study (water aerobic training program using 55 min sessions 3 days per week) with 40 hypertensive men (grade 1 and 2), a reduction of SBP of 11.71 mm Hg and of DBP of 5.9 mm Hg compared to baseline was observed [11]. It appears that the exercise component is even more important than immersion; at least, this is what a randomized controlled study with 52 post-menopausal hypertensive women, which found no difference between the effect of water-based exercise compared to land-based exercise suggests [26].

### Complex therapies

It may also be necessary to combine ITW with more complex therapies such as spa therapies. A 3-week Zelen double consent randomized controlled trial (*N* = 257) in France [27] found that a complex spa therapy with ITW (daily bubble bathing, water manual massages, mud therapy, water pool exercise, drinking of mineral water, nutrition and physical activity counseling) significantly reduced BMI (mean BMI loss after 14 months follow-up was 1.91 kg/m^2^ [95 % CI: 1.46; 2.35]; *P* < 0.001). In a randomized, controlled trial from Poland [28] 50 overweight and obese patients (BMI = 43.8) presented a significant weight reduction with an average loss of 7 % of their initial weight and other CVRF such as total cholesterol, LDL-cholesterol, triglycerides and blood glucose after a 3-week spa therapy consisting of 15 daily mineral (4 % NaCl) thermal baths (31.0 °C) in a swimming pool and mud packs and a 1000 kcal diet. Similar results were shown in a 3-week non-randomized, controlled trial (*N* = 199) with ITW as part of complex spa therapy (cardiopulmonary endurance training, individually adjusted diet, education programme on CVRF and physical and balneological treatment related to other diseases). The spa therapy significantly reduced BMI (*P* < 0.001), mean arterial BP (*P* = 0.002), apolipoprotein B (*P* = 0.015), fasting insulin (*P* = 0.035) and fibrinogen (*P* = 0.950) [18].

In our study, ITW was combined with written information about a healthier lifestyle. We expected high motivation or adherence to a healthier lifestyle. However, this intervention did not seem to be intense enough. This may be explained by the fact that the participants relied completely on the effects of ITW or the relaxation technique. Another explanation for the minimal effect over time in all three treatment groups may be that unspecific effects such as regression to the mean were minimized in our trial, as there was a 1-week gap between the time of inclusion and baseline. One trial in Germany investigating spa therapy including ITW in mineral water found that 50 % of the moderate effects on CVRF happened during the first day of treatment [29].

#### Study limitation

The strengths of our study include the randomized, controlled design, blinded statistical analyses and data management. The outcome parameters were measured independently and were not influenced by the outcome assessor (JN). Limitations include the relatively small study size, the absence of supervision in the ITW groups (Bath1, Bath2) and the control group (relaxation), and, most importantly, the absence of blinding in the treatment groups, which is inherent and inevitable, due to the active participation required by the study participants. As recruitment via physicians (primary care centers), hospitals and pharmacies was difficult (*n* = 4 patients), the majority of patients was recruited via public announcement. Therefore we had to screen many non-hypertensive patients, respectively well-controlled patients, who hoped to reduce their medication during the trial. This resulted in >1000 screened patients, however, from our point of view not resulting in a relevant bias. The drop-out rate was relatively low, taking into account the intensive treatment with four ITW-sessions per week. Nevertheless, it was still about 10 % (6/59). Due to the small number of participants, subgroup analyses were not performed (i.e. different antihypertensive medication vs. no medication).

#### Generalizability

Although external validity may be restricted due to the population selected to participate in clinical studies, the population studied here can be regarded as representative of routine clinical practice, including patients with and without antihypertensive medication [30]. We had only few exclusion criteria and 63 % (37/59) of our study population comprised regularly working people (>3 h/d). We had expected a higher percentage of non-working people (pensioners, housewives) as four baths a week are quite time-consuming.

#### Clinical implications

ITW alone cannot be recommended as adjuvant therapy to reduce CVRF in hypertensive patients grade 1 and 2. As shown in other studies, it may be important to combine ITW with aquatic exercise, which seems to be effective in at least reducing elevated BP, or other balneological therapies.

## Conclusions

It seems eminently important to combine ITW with other treatments for lifestyle change such as complex balneotherapy, diet, physical activity, or at least appropriate counseling. Further evaluation in rigorously designed clinical studies will be necessary to validate the impact of balneotherapy on CVRF.

## Abbreviations

ACEI, angiotensin-converting enzyme inhibitor; ANCOVA, analysis of covariance; ANOVA, analysis of variance; ARB, angiotensin receptor blocker; BMI, body mass index; BP, blood pressure; CCB, calcium channel blocker; CRP, c-reactive protein; CVD, cardiovascular disease; CVRF, cardiovascular risk factors; DBP, diastolic blood pressure; HDL, high-density lipoprotein; ITT, intention-to-treat; ITW, immersion in thermoneutral water; LDL, low-density lipoprotein; SBP, systolic blood pressure

## Additional files

Additional file 1: Table S1. Mineral content of the therapeutic pools (mg/L). (PDF 39 kb) Additional file 2: Table S2. Antihypertensive medication (intention-to-treat population, *N* = 59). (PDF 52 kb)
