# Supplementary material for: Outcomes from a three-arm randomized controlled trial of frequent immersion in thermoneutral water on cardiovascular risk factors
Source: BMC Complement Altern Med. 2016 Jul 27;16:250. doi: 10.1186/s12906-016-1241-7 (PMC4964283; doi:10.1186/s12906-016-1241-7)
Supplement: Additional file 1: Table S1. — Mineral content of the therapeutic pools (mg/L). (PDF 39 kb) [file 12906_2016_1241_MOESM1_ESM.pdf]

Suppl. Table S1: Mineral content of the therapeutic pools (mg/L)

Cations	Bad Krozingen	Freiburg	Bad Bellingen
Ca^{+2}	821	637	355
Na^{+2}	301	403	1060
Mg^{+2}	98	149	118
K^{+}	73	70	31
**Anions**			
SO_4^{-2}	1710	2250	152
HCO_3^{-}	1208	866	845
Cl^{-}	115	91	2040
CO_2	1245	691	1010
