# Supplementary material for: Outcomes from a three-arm randomized controlled trial of frequent immersion in thermoneutral water on cardiovascular risk factors
Source: BMC Complement Altern Med. 2016 Jul 27;16:250. doi: 10.1186/s12906-016-1241-7 (PMC4964283; doi:10.1186/s12906-016-1241-7)
Supplement: Additional file 2: Table S2. — Antihypertensive medication (intention-to-treat population, N = 59). (PDF 52 kb) [file 12906_2016_1241_MOESM2_ESM.pdf]

Suppl. Table S2: Antihypertensive medication (intention-to-treat population, N = 59)

							Antihypertensive medication				
Group	ACEI	ARB	Diuretics	CCB	Beta blockers	Others	1	2	3	> 3	N (%)
Bath1 N = 19	5	4	5	6	4	1	3	4	3	1	11 (57.9%)
Bath2 N = 21	3	3	2	2	2	-	4	2	-	1	7 (33.3%)
Control N = 19	6	5	5	4	4	1	8	5	1	1	15 (78.9%)
											N = 33 (55.9%)

ACEI: angiotensin-converting enzyme inhibitor;

ARB: angiotensin receptor blocker;

CCB: calcium channel blocker
